# Assessing manifestations of bossing in the context of personality traits of business managers

**DOI:** 10.3389/fpsyg.2023.1115310

**Published:** 2023-03-14

**Authors:** Robert Stefko, Zuzana Birknerova, Lucia Zbihlejova, Lubomir Nebesky

**Affiliations:** ^1^Department of Marketing and International Trade, Faculty of Management and Business, University of Presov, Presov, Slovakia; ^2^AMBIS University, Prague, Czechia; ^3^Department of Managerial Psychology, Faculty of Management and Business, University of Presov, Presov, Slovakia; ^4^Department of Intercultural Communication, Faculty of Management and Business, University of Presov, Presov, Slovakia; ^5^Department of Economics and Finance, Bratislava University of Economics and Management, Bratislava, Slovakia; ^6^Ministry of Education, Science, Research and Sport of the Slovak Republic, Bratislava, Slovakia

**Keywords:** business manager, bossing, personality traits, BOSSm18 methodology, B5 methodology

## Abstract

**Introduction:**

Abusive supervision or bossing represents a specific form of mobbing as a long-term, systematic negative action by superiors toward subordinates.

**Methods:**

From the point of view of the operationalization of this construct, the original BOSSm18 methodology is presented in the paper in the context of the B5 methodology, which enables specification of personality traits in terms of the original Big Five concept.

**Results:**

Based on the research dataset of 636 business managers, the paper presents the results of the basic psychometric parameters of the methodology and the content specification of the extracted factors. The research findings support a multidimensional understanding of the bossing construct.

**Discussion:**

The limiting factors of the interpretation and generalization of the results relate to the consideration of cultural contexts and situational conditions of perception of bossing manifestations.

## 1. Introduction

The impact of the work environment is multidimensional. It includes both physical influences and various psychological aspects. In this context, the negative impact of social-psychological factors also comes to the fore (Frankovský et al., 2019). These factors are detrimental to productivity and conflict with the interests of the organization. They also have a negative impact on the psychological and physical health of individuals (Bennett, 2000; Hafidz, 2012; Howladar et al., 2018; Horvathova et al., 2020). When studying undesirable behavior in the workplace, different authors use different terminology, e.g., workplace deviance (Robinson and Bennett, 1995), counterproductive work behavior (Spector and Fox, 2002), as well as antisocial behavior, bad behavior in the organization, unethical behavior (e.g., Tomkova et al., 2021), or dysfunctional behavior. In this context, terms such as workplace bullying, mobbing, and bossing appear (Einarsen et al., 2011).

Hirigoyen (2000) introduces workplace bullying as a phenomenon which dates back to the very beginnings of work as such, but it was only in the late 20th century when it started becoming a major cause of tension increase, labor productivity reductions, and work absence caused by mental disorders naturally generated by it. Bullying can affect anyone anywhere but, in some industries, where it has the most suitable conditions to develop, its occurrence is more frequent. In terms of the behavior of chief executive officers (CEO), literature provides insights into how this behavior relates to the performance of an organization (e.g., Bandiera et al., 2020), risk-taking (e.g., Bernile et al., 2017), its sustainability (e.g., Abatecola and Cristofaro, 2019), or organizational culture (e.g., Tsui et al., 2006). Less information is available on the negative or undesirable forms of the behavior of a CEO, which presents a challenge for conducting a research study on this particular matter. In this sense, the presented research is aimed at assessing manifestations of abusive supervision or bossing in the context of personality traits of business managers.

The research is divided into several parts. Firstly, the theoretical framework of bossing and personality traits of CEO and business managers are presented. The section is followed by the research on the studied issue, discussion, the limiting factors, and a few future directions. According to the research findings it is possible to use the presented study theoretically as well as practically on all levels of (business) management as the utilized methodologies may serve as tools for the HR management of any company to hire personnel for managerial positions.

## 2. Bossing and personality traits of managers

Safina and Podgornaya (2014) point out that bullying occurs when an individual is oppressed by another individual or by a group of people. The authors define bullying as a moral and often physical persecution at the workplace (i.e., mental terror, pressure, or harassment). In this context, Droppa et al. (2018) add that bullying is a demonstration of the superiority of one person over another, which can happen for various reasons. Yaman (2009), Senol et al. (2015), and others describe mobbing as a horizontal form of workplace bullying which occurs between two or more people working on the same position within the hierarchy of an organization from the lowest working positions to the top management (i.e., just as a production worker can mob another production worker, a top manager can mob another top manager). When the initiator of aggression is a senior executive, this kind of bullying is called abusive supervision or bossing. It is a type of vertical abuse directed from a higher-level position to a lower-level position within the organizational hierarchy (i.e., supervisor/manager can use bossing on the subordinates, or a top manager can use it to abuse a lower-level type of a manager). Arnejčič (2016) also reports that bossing is a form of “vertical wall” mobbing, occurring when a person tries to denigrate his or her subordinate. As opposed to bossing (downward bullying by supervisors), Oberhofer (2018) uses the term staffing (upward bullying by subordinates) and stresses that both these terms are typical primarily in German-speaking regions of Europe. Zikic et al. (2013) agree with this theory, stating that vertical form of mobbing (abusive supervision, bossing) occurs when a supervisor mobs a subordinate.

Bossing is a negative socio-psychological manifestation in the workplace representing a very specific form of mobbing, in which the initiator of the psychological pressure is the superior (Borská, 2005). When defining bossing, regular and long-term negative interaction between the superior and their employees is assessed. The main feature of bossing is systematic-ness and long-term-ness (Birknerová et al., 2010). Exposure to such systematic, negative, and counter-social acts can be seen as a type of psychological siege. Leymann (1990) described it as a psychological terror that can lead to serious physical, psychological, and social problems. In terms of bossing, the employees often find themselves in a situation that threatens them much more than any other. It is not only about social contacts and a sense of personal happiness, but also about professional identity, career, and, from an economic point of view, often about one’s own existence (Huberová, 1995; Luu, 2019; Wongleedee, 2020). According to Olšovská (2013), this is a conscious reduction of dignity, intimidation of an employee by a superior, resulting in mental, moral, physical, or social harm. The aim of bossing is to disrupt the working atmosphere of an individual, mostly due to emphasizing their hierarchical position in the company, maintaining power, or for various personal interests.

Bossing can influence the long-term cooperation of the working teams as well as the overall performance of an organization, and it significantly lowers the employees’ focus on meeting the goals of their organization, reducing the quality of their work (Jenčo et al., 2018). Zhao (2018, p. 154) adds that “abusive supervision can not only directly reduce the performance of subordinates, but also can have a negative impact on performance by reducing the identity of subordinates to leadership.” Bossing is an apparent cause of the decrease in the performance of employees and therefore performance of an organization as a whole, particularly in terms of its sustainability. However, Lu (2013) argue that bossing may quite contrarily inspire job passion among employees and thus improve the overall performance of an organization. In this context it is therefore crucial to distinguish between bossing as abusive supervision, which leads to performance decrease, and bossing as requiring consistent performance of duties, which leads to performance increase.

A leader has other motives for bullying and uses other strategies than a co-worker. Among their basic motives is jealousy of a skilled worker and fear of losing their position, creating pressure on the subordinate worker to enforce their obedience, efforts to expel this person from the working team, or from the workplace as such (Olšovská, 2013). Anger at the organization is also notable, as well as hatred of the superiors, and negative personal qualities of the leader, which get an opportunity to manifest themselves at the moment when the individual acquires power or influence (Camps et al., 2016; Wilson and Nagy, 2017).

Cloutier et al. (2015) state that the leadership of a company must understand their abilities and their impact on their employees and, according to these, choose appropriate forms of communication, motivation, and teamwork. Successful is the one characterized by personality traits and qualities. Over the past years, the following basic qualities of a CEO have been gaining in importance (Den Hartog et al., 2007; Mižíčková et al., 2007):

- *Dynamism*—willingness to take initiative, energy and strive to meet goals.
- *Motivation*—the need to lead and influence others.
- *Integrity*—honesty and truthfulness.
- *Self-confidence*—determination, assertiveness, and confidence.
- *Intelligence*—the ability to work with complex information.
- *Knowledge*—about the company, industry, workplace, technology.

Brown and Trevino (2006) add that the top managers, who can be described as ethical leaders, are characterized by personality traits such as honesty, integrity, and trustworthiness. They tend to engage in positive behaviors such as exemplary behavior, fair, and respectful treatment of people, and open discussion of problematic issues. Leaders with these qualities become successful managers respected by their colleagues and subordinates.

According to Bainbridge et al. (2022), a comprehensive organizing framework for psychological personality trait scales is represented by the Big Five concept. This concept is a model used primarily in psychology to describe a person’s personality. Based on Big Five, each person’s personality can be described through five characteristics or traits, namely neuroticism, extraversion, openness, agreeableness, and conscientiousness, using complex statistical methods. Beginnings of this model date back to Fiske (1949), and have since been further developed and amended by many other authors (e.g., Janovská, 2011; Ziaran et al., 2021). Camps et al. (2016) used this particular method in their research, connecting it with the issue of abusive supervision (i.e., bossing). Personality of an employee is in the workplace environment linked to the personality predispositions of the superior. In the context of the above findings, the presented research focuses on the analysis of links between the selected personality traits of managers and self-assessment of tendencies to bossing manifestations.

## 3. Research

The main objective of the presented research was to enrich knowledge in the field of operationalization and conceptualization of the issue of bossing based on the assessment of the tendency to its manifestations in the context of the personality traits of managers. In terms of this objective, the following research questions were formulated:

1. Is it possible to create a combined model of the factors of the BOSSm18 questionnaire and the personality dimensions of the B5 questionnaire?
2. Are there any statistically significant links between the factors of the BOSSm18 questionnaire and the personality dimensions of the B5 questionnaire?

### 3.1. Research sample

The research dataset consisted of 636 business managers. In terms of gender, there were 392 (61.6%) women and 244 (38.4%) men in the research sample. The average age of the respondents was 30.7 years (SD = 10.237 years), the age range was from 20 to 58 years. The average length of practice was 10.5 years (SD = 10.090 years), the range of years of practice was from 1 to 37 years. The selection of respondents can be characterized as occasional (intentional in terms of narrowing the data to business managers only). The data were collected in the period from September 2021 to May 2022. According to Finstat, there are 252,369 registered companies in the Slovak Republic in 2021. A total of 1,650 respondents were contacted *via* questionnaire based on availability. Of the total number of questionnaires distributed, the return was at the level of approx. 87%, which represented 1,435 questionnaires. From the initial number of all companies operating in Slovakia in 2021, with a marginal error of 5% and a 95% confidence interval, the total number of necessary respondents was calculated at the level of 636. These 636 respondents, who made up the research set, were selected from 1,435 returned questionnaires using a random number generator. Applying this procedure allows us to claim that the research sample is representative.

### 3.2. Research methods

In the research, two questionnaires were applied and administered to managers in bulk and anonymously. These questionnaires enabled operationalization of the variable “bossing” (three factors: Communication-Aimed Bossing, Work-Aimed Bossing, Psyche-Aimed Bossing), and the variable “personality traits” (five factors: Extraversion, Agreeableness, Conscientiousness, Neuroticism, and Openness).

The BOSSm18 questionnaire (Birknerová et al., 2022) was used as the original method in the presented research. It is constructed on the basis of a multidimensional understanding of this construct and contains 18 items that reveal a tendency toward negative forms of behavior toward the employee on the part of the superior. Respondents assess the individual items on a scale from 1 to 5 (1—completely disagree; 2—rather disagree; 3—neither agree nor disagree; 4—rather agree; and 5—completely agree). Through Exploratory factor analysis, three factors were extracted: Communication-Aimed Bossing (CAB), Psyche-Aimed Bossing (PAB), and Work-Aimed Bossing (WAB). The extracted factors explain 66.804% of the variance. The appropriateness of using factor analysis is confirmed by the Kaiser-Mayer-Olkin measure of Sampling Adequacy (0.928), Bartlett’s test of sphericity (χ2 = 7593.887, df = 153, *p* = 0.000) and leads to the conclusion that the factor analysis was suitable for this particular dataset. The reliability of the methodology was determined by assessing Cronbach’s alpha coefficients:

- BOSSm18 α = 0.932.
- CAB α = 0.864.
- PAB α = 0.922.
- WAB α = 0.855.

Table 1 presents the content specification of the factors.

**Table 1:** Description of the factors of the BOSSm18 methodology.

Factor	No. of items	Description
Communication-aimed bossing (CAB)	6	The factor assesses: to what extent the superior would allow the subordinate to comment on criticism; provide them with access to information; invite them to team meetings and operational meetings; provide them with a turn to speak; and communicate with them
Psyche-aimed bossing (PAB)	8	The factor assesses: the extent to which the superior would verbally attack the subordinate; spread rumors and intrigues about them; ignore their opinion; mock them; assign them inappropriate work; and over-control them
Work-aimed bossing (WAB)	4	The factor assesses: to what extent the superior would damage the subordinate’s things and work results; threaten with violence; bother them; and question their mental state

Personality traits of the respondents were measured by the B5 questionnaire, which is an abbreviated version of the Big Five by Janovská (2011). It consists of 40 expressions-qualities that describe the personality traits of individuals. They are evaluated on an 8-point scale (1—completely inaccurate; 2—very inaccurate; 3—relatively inaccurate; 4—slightly inaccurate; 5—slightly accurate; 6—relatively accurate; 7—very accurate; and 8—completely accurate). The qualities are arranged independently in the questionnaire. They fall within 5 subscales, measuring 5 personality factors (dimensions):

- *Extraversion* (cheerful, extroverted, alert, enthusiastic, friendly, and sociable).
- *Agreeableness* (kind-hearted, gentle, sensitive, accommodating, helpful, amiable, compassionate).
- *Conscientiousness* (conscientious, thorough, orderly, humble, meticulous, reliable, and self-disciplined)
- *Neuroticism* (tense, restless, unbalanced, worried, oversensitive, nervous, and insecure).
- *Openness* (unconventional, abstract thinking, probing, calculated, thoughtful, interested, searching, and inventive).

The respondents achieved the highest average score calculated as a simple sum of the questionnaire items in terms of the defined model in the Work-Aimed Bossing with a value of 11.563 ± 0.411; subsequently, in the Communication-Aimed Bossing factor with the achieved value of 9.874 ± 0.349; then in the Psyche-Aimed Bossing factor with an average value of 4.524 ± 0.125. Based on the Skewness and Kurtosis testing, the distribution of the data can be considered normal.

For the purposes of our research, we chose the maximum likelihood (ML) method. Selection of the CFA method was based on the results of available research studies (Rhemtulla et al., 2012; Xia and Yang, 2019), which accept the application of classic cut-off estimators by the ML method even in the case of ordinal variables if they have at least 5 response categories. Then they can be regarded as interval variables.

### 3.3. Research results

**Figure 1:**
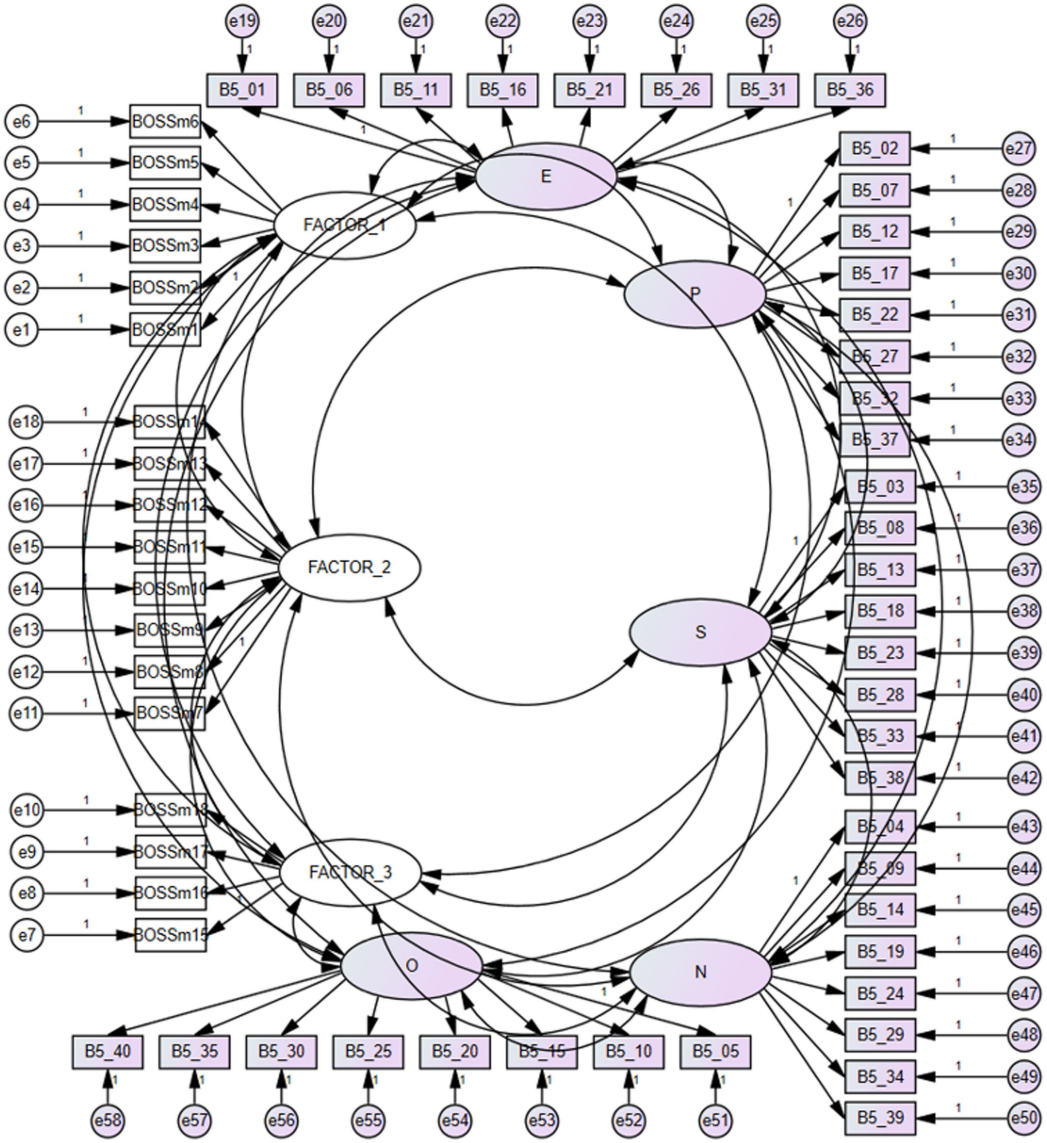
The theoretical model of the links between BOSSm18 and B5FACTOR_1 = CAB (BOSSm18), FACTOR_2 = PAB (BOSSm18), FACTOR_3 = WAB (BOSSm18). E, extraversion (B5); P, agreeableness (B5); S, conscientiousness (B5); N, neuroticism (B5); O, openness (B5).

The main objective of the research was to assess the interrelationships between the three extracted factors of the BOSSm18 questionnaire (FACTOR 1 to FACTOR 3) and the five personality dimensions resulting from the B5 questionnaire. The approach analyzes whether the theoretical model (Figure 1) shows the consistency of the data obtained through research.

Table 2 points to the fact that the calculated model indicators are within the range of acceptable and perfect indicators. None of these indicators were outside the recommended intervals. It is clear from Table 2 that the recommended indices evaluating the factor model (Figure 1) are acceptable and authorize us to state that the created hypothetical model presents a good degree of agreement with the real data and is applicable in this form.

**Table 2:** CFA fit indices for the whole BOSSm18 model.

Fit indices used	Perfect fit indices	Acceptable fit indices	CFA results	References
*χ2/df*	0 ≤ *χ2/df* ≤ 2	2 ≤ *χ2/df* ≤ 3	1.667	Hu and Bentler (1998)
*GFI*	0.95 ≤ *GFI* ≤ 1.00	0.90 ≤ *GFI* ≤ 0.95	0.923	Marsh et al. (1988), Jöreskog and Sörbom (1993), Schermelleh-Engel et al. (2003)
*AGFI*	0.90 ≤ *AGFI* ≤ 1.00	0.85 ≤ *AGFI* ≤ 0.90	0.905	
*CFI*	0.95 ≤ *CFI* ≤ 1.00	0.90 ≤ *CFI* ≤ 0.95	0.967	Bentler and Bonett (1980), Bentler (1980), Marsh et al. (2006)
*NFI*	0.95 ≤ *NFI* ≤ 1.00	0.90 ≤ *NFI* ≤ 0.95	0.924	
*TLI*	0.97 ≤ *TLI* ≤ 1.00	0.95 ≤ *TLI* ≤ 0.97	0.958	
*RMSEA*	0.00 ≤ *RMSEA* ≤ 0.05	0.05 ≤ *RMSEA* ≤ 0.08	0.033	Browne and Cudeck (1993), Byrne and Campbell (1999), Hu and Bentler (1999), Schermelleh-Engel et al. (2003)
*SRMR*	0.00 ≤ *SRMR* ≤ 0.05	0.05 ≤ *SRMR* ≤ 0.10	0.0417	

χ2, Chi-square; df, degrees of freedom; GFI, goodness of fit index; AGFI, adjusted goodness of fit index; CFI, comparative fit index; NFI, The Bentler-Bonett normed fit index; TLI, Tucker-Lewis coefficient; RMSEA, root mean square error of approximation; SRMR, standardized root mean square residual.

We noted the existence of links between the factors of the BOSSm18 questionnaire, links between the items of the questionnaire, as well as links between the items of the BOSSm18 questionnaire and B5.

It is clear from Table 3 that the Openness dimension of B5 is not influenced by its items. Table 4 confirms that Openness is not related to any bossing factors. In the table we present the analysis of the dispersion of individual latent variables (Factor 1: Openness) in terms of statistical significance in the model (Figure 1).

**Table 3:** Regression weights and standardized errors of the combined model of BOSSm18 and B5.

Relationship	Estimate	Std. estimate	Std. error	*t*-statistic	*p*-value
BOSSm1 <−-- CAB	1.000	0.584			
BOSSm2 <−-- CAB	0.934	0.587	0.088	10.551	0.000*
BOSSm3 <−-- CAB	1.130	0.797	0.074	15.203	0.000*
BOSSm4 <−-- CAB	1.226	0.773	0.082	15.024	0.000*
BOSSm5 <−-- CAB	1.241	0.795	0.094	13.194	0.000*
BOSSm6 <−-- CAB	1.206	0.748	0.091	13.278	0.000*
BOSSm15 <−-- WAB	1.000	0.879			
BOSSm16 <−-- WAB	0.447	0.602	0.030	15.129	0.000*
BOSSm17 <−-- WAB	0.516	0.594	0.034	15.003	0.000*
BOSSm18 <−-- WAB	0.740	0.742	0.038	19.604	0.000*
BOSSm7 <−-- PAB	1.000	0.719			
BOSSm8 <−-- PAB	1.114	0.790	0.061	18.308	0.000*
BOSSm9 <−-- PAB	1.170	0.811	0.056	20.834	0.000*
BOSSm10 <−-- PAB	1.145	0.812	0.056	20.443	0.000*
BOSSm11 <−-- PAB	0.940	0.821	0.046	20.384	0.000*
BOSSm12 <−-- PAB	0.948	0.648	0.055	17.391	0.000*
BOSSm13 <−-- PAB	0.991	0.715	0.055	17.910	0.000*
BOSSm14 <−-- PAB	0.919	0.759	0.105	8.715	0.000*
B5_01 <−-- Extraversion	1.000	0.747			
B5_06 <−-- Extraversion	0.157	0.102	0.061	2.566	0.010*
B5_11 <−-- Extraversion	0.634	0.520	0.046	13.926	0.000*
B5_16 <−-- Extraversion	1.168	0.846	0.051	22.806	0.000*
B5_21 <−-- Extraversion	1.101	0.868	0.053	20.719	0.000*
B5_26 <−-- Extraversion	0.882	0.687	0.049	17.999	0.000*
B5_31 <−-- Extraversion	1.292	0.908	0.053	24.529	0.000*
B5_36 <−-- Extraversion	0.713	0.579	0.047	15.217	0.000*
B5_02 <−-- Agreeableness	1.000	0.837			
B5_07 <−-- Agreeableness	1.053	0.881	0.036	29.047	0.000*
B5_12 <−-- Agreeableness	0.942	0.726	0.042	22.323	0.000*
B5_17 <−-- Agreeableness	1.000	0.825	0.037	27.121	0.000*
B5_22 <−-- Agreeableness	0.899	0.790	0.036	25.105	0.000*
B5_27 <−-- Agreeableness	0.916	0.798	0.036	25.563	0.000*
B5_32 <−-- Agreeableness	1.083	0.897	0.035	31.380	0.000*
B5_37 <−-- Agreeableness	1.072	0.851	0.038	28.353	0.000*
B5_03 <−-- Conscientiousness	1.000	0.738			
B5_08 <−-- Conscientiousness	1.056	0.733	0.045	23.365	0.000*
B5_13 <−-- Conscientiousness	0.335	0.205	0.073	4.590	0.000*
B5_18 <−-- Conscientiousness	0.715	0.468	0.059	12.037	0.000*
B5_23 <−-- Conscientiousness	0.941	0.582	0.064	14.790	0.000*
B5_28 <−-- Conscientiousness	1.080	0.707	0.055	19.493	0.000*
B5_33 <−-- Conscientiousness	1.179	0.875	0.052	22.633	0.000*
B5_38 <−-- Conscientiousness	1.086	0.797	0.052	20.936	0.000*
B5_04 <−-- Neuroticism	1.000	0.748			
B5_09 <−-- Neuroticism	0.498	0.357	0.054	9.225	0.000*
B5_14 <−-- Neuroticism	0.715	0.462	0.064	11.230	0.000*
B5_19 <−-- Neuroticism	0.594	0.454	0.056	10.559	0.000*
B5_24 <−-- Neuroticism	0.502	0.366	0.059	8.460	0.000*
B5_29 <−-- Neuroticism	0.498	0.357	0.059	8.487	0.000*
B5_34 <−-- Neuroticism	1.089	0.746	0.065	16.826	0.000*
B5_39 <−-- Neuroticism	0.260	0.202	0.048	5.432	0.000*
B5_05 <−-- Openness	1.000	0.043			
B5_10 <−-- Openness	15.406	0.631	15.333	1.005	0.315
B5_15 <−-- Openness	15.058	0.658	14.997	1.004	0.315
B5_20 <−-- Openness	12.431	0.543	12.383	1.004	0.315
B5_25 <−-- Openness	13.187	0.604	13.133	1.004	0.315
B5_30 <−-- Openness	13.182	0.605	13.122	1.005	0.315
B5_35 <−-- Openness	3.192	0.131	3.328	0.959	0.338
B5_40 <−-- Openness	11.218	0.564	11.175	1.004	0.315

* Significant at the level of significance α = 0.05,

Estimate, estimate; Std. Estimate, standardized regression weight; Std. error, standard error; t, *t*-statistic; p, probability level.

**Table 4:** Analysis of variance of latent extracted variables of the combined model BOSSm18 and B5.

	Estimate	Std. error	*F*-statistic	*p*- value
CAB	0.377	0.048	7.888	0.000*
WAB	0.254	0.020	12.852	0.000*
PAB	0.360	0.034	10.451	0.000*
Extraversion	1.587	0.141	11.291	0.000*
Agreeableness	2.049	0.150	13.662	0.000*
Conscientiousness	1.465	0.134	10.945	0.000*
Neuroticism	1.716	0.165	10.429	0.000*
Openness	0.005	0.011	0.501	0.616

*Significant at the level of significance α = 0.05.

Table 4 illustrates that the B5 questionnaire dimension of Openness is not significant at the level of significance α = 0.05. It means that business managers, who assessed themselves as open, manifest less tendency to boss their subordinates. However, it is crucial here to accentuate that there is rather a non-existing relationship between these two variables, rather than an existing negative one. Table 5 analyzes the mutual relationship between the latent variables (Factor 1 ... Openness) in the form of correlations. Between the latent variables of the BOSSm18 questionnaire (between themselves), the values of the correlation coefficients are higher than in the other cases. This is due to the consistency of the test itself. The table shows the relationships between the personality dimensions of the B5 questionnaire.

**Table 5:** Links between the latent variables of the combined model of BOSSm18 and B5.

Relationship	Covariance				Correlation
	Estimate	Std.error	*t*-statistic	*P*-value	Estimate
CAB <−-> WAB	0.167	0.019	8.608	0.000*	0.540
CAB <−-> PAB	0.278	0.028	10.021	0.000*	0.755
CAB <−-> Extraversion	−0.089	0.024	−3.661	0.000*	−0.115
CAB <−-> Agreeableness	−0.114	0.029	−3.952	0.000*	−0.130
CAB <−-> Conscientiousness	−0.071	0.027	−2.631	0.009*	−0.096
CAB <−-> Neuroticism	0.135	0.033	4.132	0.000*	0.167
CAB <−-> Openness	−0.005	0.005	−0.940	0.347	−0.105
WAB <−-> PAB	0.230	0.019	12.190	0.000*	0.761
WAB <−-> Extraversion	−0.065	0.020	−3.321	0.000*	−0.102
WAB <−-> Agreeableness	−0.075	0.023	−3.269	0.001*	−0.104
WAB <−-> Conscientiousness	−0.051	0.022	−2.354	0.019*	−0.084
WAB <−-> Neuroticism	0.034	0.026	1.325	0.185	0.052
WAB <−-> Openness	0.000	0.001	−0.308	0.758	−0.012
PAB <−-> Extraversion	−0.111	0.024	−4.695	0.000*	−0.147
PAB <−-> Agreeableness	−0.133	0.028	−4.759	0.000*	−0.155
PAB <−-> Conscientiousness	−0.099	0.027	−3.698	0.000*	−0.137
PAB <−-> Neuroticism	0.082	0.031	2.659	0.008*	0.104
PAB <−-> Openness	−0.005	0.005	−0.942	0.346	−0.105
Extraversion <−-> Agreeableness	1.688	0.126	13.373	0.000*	0.936
Extraversion <−-> Conscientiousness	0.976	0.088	11.062	0.000*	0.640
Extraversion <−-> Openness	0.070	0.070	0.994	0.320	0.755
Agreeableness <−-> Conscientiousness	1.224	0.103	11.855	0.000*	0.707
Agreeableness <−-> Openness	0.082	0.083	0.999	0.318	0.784
Conscientiousness <−-> Openness	0.073	0.073	1.000	0.317	0.826
Extraversion <−-> Neuroticism	−0.381	0.066	−5.770	0.000*	−0.231
Agreeableness <−-> Neuroticism	−0.515	0.075	−6.852	0.000*	−0.275
Conscientiousness <−-> Neuroticism	−0.278	0.068	−4.054	0.000*	−0.175
Neuroticism <−-> Openness	−0.031	0.031	−0.999	0.318	−0.321

* Significant at the level of significance α = 0.05.

Estimate, estimate; Std. Estimate, standardized regression weight; Std. error, standard error; *t*, *t*-statistic; *p*, probability level.

Table 5 shows the interrelationships between the three factors of the BOSSm18 questionnaire and the personality dimensions of the B5 questionnaire. No relationship of any latent variable with Openness is significant. Bossing factors are positively related to the Neuroticism dimension, and at the same time Neuroticism does not have a significant relationship with the factor Work-Aimed Bossing (WAB). On the other hand, the other (except for Openness) personality dimensions of the B5 questionnaire are linked to Bossing through negative ties. The friendlier a manager is, the less inclined he or she is to practice or apply bossing in all its dimensions (Factors 1, 2, and 3).

Based on the above analyses, the two formulated research questions have been answered:

1. It is possible to create a combined model of the factors of the BOSSm18 questionnaire and the personality dimensions of the B5 questionnaire.
2. There are certain statistically significant links between the factors of the BOSSm18 questionnaire and the personality dimensions of the B5 questionnaire.

### 3.4. Common method bias minimization

When analyzing covariance and mean structures, two critically important assumptions associated with structural equation modeling (SEM) are the requirement that the data be continuous and have a multidimensional normal distribution. The requirement of continuity is also met for ordinal variables, if we proceed from the research results of Rhemtulla et al. (2012) and Xia and Yang (2019), which accept the application of classic cut-off estimators by the ML method also in the case of ordinal variables, if they have at least 5 response categories, when they can be thought of as interval variables. These basic assumptions are linked to the theory of large samples in which SEM is incorporated. More precisely, they derive from the approach used in parameter estimation using the SEM methodology. Usually either a maximum likelihood (ML) method or a method based on the theory of generalized least squares (GLS) estimation. The key idea behind the bootstrap technique is that it allows multiple sub-samples to be created from the original database. The importance of this procedure is that one can then examine the parameter distributions with respect to each of these generated samples. These distributions serve as bootstrap sampling distributions, which technically work in the same way as the sampling distribution generally associated with parametric inferential statistics. Unlike traditional statistical methods, the bootstrapping sampling distribution is specific and allows comparison of parametric values of replicate samples that have been drawn (with replacement) from the original sample.

In general, the main advantage of bootstrapping is that it allows the stability of parameter estimates to be assessed and thus estimated with greater precision. Within the more specific context of SEM, the bootstrap procedure provides a mechanism for dealing with situations where statistical assumptions about sample size and multivariate normality may not hold (Yung and Bentler, 1996). Perhaps, the strongest advantage of bootstrapping in SEM is its “automatic” refinement based on standard asymptotic theories so that bootstrapping can be used even for samples with small (but not extremely small) sizes (Yung and Bentler, 1996). However, bootstrapping has its limitations. If a new distribution is generated from the original sample using bootstrap, it is important that the original sample is representative of the population. If this assumption is violated, i.e., the original sample is not representative, the application of the bootstrap procedure will lead to misleading results (Zhu, 1997). Second, Yung and Bentler (1996) noted that for the bootstrap to work under covariance structure analysis, the assumption of independence and equal distribution of unit observations must be met. They argued that such an assumption is necessary for any justification of surrogate sampling from a reproduced bootstrap correlation matrix. Third, the success of bootstrap analysis depends on the degree of consistency that represents the behavior of the statistic of interest when samples are drawn from an empirical distribution and when they are drawn from the original population (Bollen and Stine, 1992).

As part of the analysis, the first step was to select a sample from the original data matrix with the number of *N_b1_* = 500, using the ML estimator, with estimation of confidence intervals with deviation correction for each parameter with a 90% confidence interval. For this analyzed sample, the bootstrap estimate for the standard error is at the average level of 0.024, but with certain discrepancies between the original and bootstrapped causes, especially for items B5_05, B5_10, B5_15, B5_20, B5_25, B5_30, B5_35, and B5_40. For these items, the standard error difference between the original and the bootstrap sample is 1.557. But even this value does not cause a change in the result and the decision that Openness is significantly influenced by the aforementioned items of the research tool. For a bootstrapped sample with a population of 500, the results indicate that the parameter estimates are more or less identical to the original sample and thus we cannot expect the presence of outliers or their skew. The error estimate of the standard error of the model itself varies for individual parameters of the model in a narrow interval of 0.000 to 0.002, which represents an acceptable level. Finally, the bias values, i.e., the difference between the bootstrap average and the original estimate of the model coefficients varies depending on the error of the standard error of the model in the interval − 0.001 to 0.003 with significantly higher values for the Openness factor, where the value of bias varies in the interval − 0.006 to 0.015. In the second step, the size of the bootstrap sample with *N_b2_* = 200 was selected. Even with this size of the bootstrap sample, no significant differences in the observed parameters were demonstrated. The standard error ranged for individual items at an average level of 0.025 with a significant difference in the problematic items of the Openness factor with an average value of 1.604. The estimate of the error of the standard error of the model varies in a narrow interval of 0.000 to 0.003 for individual parameters of the model, which represents an acceptable level. Finally, the bias values ranged from −0.003 to 0.005 with significantly higher values for the Openness factor. Based on these results, it is possible to consider the defined research sample as internally consistent without the presence of extreme values, and the results obtained using confirmatory factor analysis as correct.

## 4. Discussion

In the context of research on undesirable forms of behavior by superiors toward subordinates, it is possible to discuss several concepts, such as downward bullying (Oberhofer, 2018), vertical mobbing (Zikic et al., 2013; Arnejčič, 2016), toxic bossing (Hamilton et al., 2017), and boss syndrome (Emelander, 2011). Oberhofer (2018) states that bossing is a specific term used primarily in some European, German-speaking regions. Arnejčič (2016) categorizes bossing within the so-called vertical mobbing, which occurs when an individual slanders a subordinate employee. Zikic et al. (2013) agree with this theory and argue that vertical mobbing or bossing occurs when a subordinate is mobbed by a supervisor. Hamilton et al. (2017) present the concept of toxic bossing, which they decipher as forms of abusive behavior aimed at subordinates in the workplace. In this context, Emelander (2011) uses the more general term “boss syndrome” if superiors do not know how to treat the most important resource of the company, i.e., people.

Most research relating to the personality contexts of bossing is focused on examining the personality traits of a superior as a subject of undesirable forms of behavior (Camps et al., 2016; Wilson and Nagy, 2017). For instance, Camps et al. (2016) investigated links between the personality traits of supervisors as measured by the Big Five concept and employees’ experiences of supervisory abuse. The authors found out that while agreeableness, extraversion, openness to experience, and neuroticism of the examined supervisors were not significantly related to abusive supervision, supervisor conscientiousness and abusive supervision were revealed to have a significant positive relationship. Less research is focused on the analysis of subordinate personality traits in the context of their perception of undesirable forms of superior behavior (Brees et al., 2016). The authors examined the relationship between the personality traits of employees and their perception of abusive behavior of a superior. They found that subordinates who scored higher in negative affectivity, anger, and hostility perceived the above-mentioned manifestations of superiors.

The presented research was aimed at assessing manifestations of abusive supervision or bossing in the context of personality traits of business managers, i.e., investigating how the tendencies of managers toward these undesirable forms of behavior relate to their personality traits.

## 5. Limitations and future directions

In the presented article, attention was paid to the issue of links between personality traits and assessing manifestations of bossing. The results of the research confirmed the meaningfulness of considering the links between personality traits and assessing manifestations of bossing by top management. Analysis of the correlations between bossing factors and personality traits determined from the perspective of the Big Five concept confirmed, except for the Openness trait, the existence of statistically significant correlations. The more extraverted, friendly, and conscientious managers are, the less they tend to bully their subordinates. They are less sensitive to the occurrence of manifestations of bossing. On the contrary, it is true for neurotic managers. Openness links did not prove to be statistically significant. At this stage of the research, it is possible to consider the action of situational factors and cultural contexts as a limiting factor.

It is also crucial to point out that at present we are less likely to encounter bossing self-assessment in the literature, or other forms of bullying in the workplace by managers in terms of disposition context and personality traits. This testifies to the fact that managers did not consider undesirable forms of behavior as a tendency, a predisposition to these forms of behavior, but rather described them universally in the context of a given specific situation. We usually encounter research in which subordinates evaluate their superiors in a given context, which was not the goal in the research we presented.

At the same time, it is important to highlight the need to examine the issue of bossing in the context of personality traits on the one hand, and the conditions of occurrence of this behavior on the other. The presented concept of bossing can be considered as a dispositional approach, within which this issue is defined as a personality trait, based on which it is possible to predict the behavior trans-situationally in the sense of the superior vs. subordinate interaction.

In terms of the future directions of the presented research, it is necessary to put an emphasis on prevention. Education in this area should be key. As bossing tends to expand in businesses across the world, it would be appropriate to put periodic training on it. A good system of education, prevention, identification of bossing attributes, transparent criteria, practices, and possibly repression could help to solve this—rather complicated—problem.

## 6. Conclusion

Manifestations of bossing and mobbing are an important factor in predicting and interpreting people’s behavior in various work contexts. One of the common denominators of these manifestations is declining labor productivity, absenteeism, turnover, declining exposure, and significant economic consequences (Hirigoyen, 2000, etc). At the same time, it is necessary to point out the effect of bossing and mobbing on the psyche of each employee, with consequences also on the physical health of people. When assessing the manifestations of bossing in the behavior of superiors and drawing conclusions, it is necessary to accept a comprehensive approach that includes cultural patterns of behavior, situational conditions of this behavior, as well as personality traits of employees. It is also crucial to draw attention to the subjective perception of these behaviors.

The mentioned approach relates to the holistic concept of defining this issue. From the point of view of this concept, it is necessary to understand the economic, socio-cultural, and personality attributes as one whole, which differs from the summary of the results of the study of the individual elements of bossing. At the same time, this concept helps to define the boundary between bossing and normal, albeit harsh behavior of a superior. Since in every company, employees are used to different standards, it is sometimes very difficult to recognize what is still and what is no longer permissible and falls into manifestations of bossing (Bednař, 2016).

## Data availability statement

The raw data supporting the conclusions of this article will be made available by the authors, without undue reservation.

## Ethics statement

Ethical review and approval was not required for the study on human participants in accordance with the local legislation and institutional requirements. Written informed consent for participation was not required for this study in accordance with the national legislation and the institutional requirements.

## Author contributions

RS, ZB, and LZ contributed to conception and design of the study. RS organized the database. ZB performed the statistical analysis. LZ wrote the first draft of the manuscript. RS, ZB, LZ, and LN wrote sections of the manuscript. All authors contributed to manuscript revision and read and approved the submitted version.

## Funding

This study is an output of the science projects VEGA No. 1/0807/19 of the Scientific Grant Agency of the Ministry of Education, Science, Research and Sport of the Slovak Republic and Slovak Academy of Sciences and KEGA No. 018PU-4/2023 of the Cultural and Educational Grant Agency of the Ministry of Education, Science, Research and Sport of the Slovak Republic.

## Conflict of interest

The authors declare that the research was conducted in the absence of any commercial or financial relationships that could be construed as a potential conflict of interest.

## Publisher’s note

All claims expressed in this article are solely those of the authors and do not necessarily represent those of their affiliated organizations, or those of the publisher, the editors and the reviewers. Any product that may be evaluated in this article, or claim that may be made by its manufacturer, is not guaranteed or endorsed by the publisher.
